# 单发纯磨玻璃样结节为CT表现的原位肺腺癌：1例报道及文献复习

**DOI:** 10.3779/j.issn.1009-3419.2013.08.09

**Published:** 2013-08-20

**Authors:** 明 董, 嵩 徐, 毅 武, 懿 刘, 刚 陈, 军 陈

**Affiliations:** 300052 天津，天津医科大学总医院肺部肿瘤外科 Department of Lung Cancer Surgery, Tianjin Medical University General Hospital, Tianjin 300053, China

肺部磨玻璃影(ground-glass opaeity, GGO)是指计算机断层扫描(computered tomography, CT)图像上表现为密度轻度增加，呈局灶性云雾状密度阴影，其内的支气管及血管纹理仍可显示。近年来，随着各种诊断手段以及CT技术不断发展GGO的检出率逐渐增高，同时一些研究^[1]^显示肺部磨玻璃影(ground-glass opacity, GGO)的CT表现与早期肺癌有一定相关性。有报道^[2]^称，以GGO为表现的肺部结节，恶性率(34%)高于实性结节(7%)，混合型GGO(部分实性结节)和单纯GGO的(非实性结节)恶性率分别为64%和18%。多数单纯GGO表现的肺结节在随访过程中大小稳定，但常与非典型腺瘤样增生(atypical adenomatous hyperplasia, AAH)及原位腺癌(adenocarcinoma *in situ*, AIS)相关。我们对1例右上肺尖端磨玻璃影，随诊1年余，病灶稳定的患者，采用胸腔镜手术切除病灶，术后病理表现为原位腺癌。现结合文献，复习报告如下。

## 1 临床资料

患者，男性，53岁。因"发现右上肺结节1年余"入院。患者于2011年9月，因咳嗽咳痰1个月，于外院查胸CT，发现右上肺磨玻璃样结节，肺窗右上肺叶尖段胸膜下区可见一约1.2 cm×1.3 cm×1.4 cm大小的磨玻璃结节灶，未见明显实变区域，纵隔窗不能显示，如图 1。

**1:**
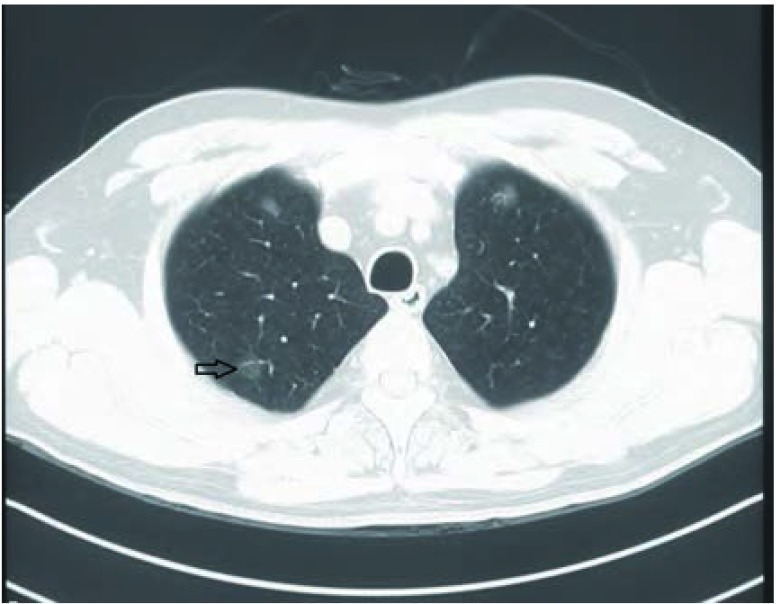
胸部CT检查。右上肺叶尖段磨玻璃样结节（箭头所指）。 CT scans of chest show the ground-glass nodule in apical segment of the superior lobe of the right lung (arrowhead).

查PET-CT提示，右肺上叶尖段磨玻璃结节灶，未见异常放射性摄取，及明显恶性征象。患者未予特殊治疗，分别于3个月、半年、1年后复查胸CT，结节大小未见明显变化，未见明显实变区域。患者入院前无咳嗽咳痰，胸闷憋气等症状，无发热胸痛，无恶心呕吐等不适。病程中精神食欲佳，二便如常，体重无明显变化。患者吸烟约20支每日，30余年。入院查体：双肺呼吸音粗，体表淋巴结未及肿大。实验室检查未见明显异常。胸部强化CT+三维重建提示：右肺上叶尖端可见磨玻璃样结节，直径约1.2 cm(图 2)。上腹强化CT、全身ECT骨扫描、颅脑强化CT未见明显异常。完善术前检查后于2013年4月11日胸腔镜下右肺上叶尖端结节楔形切除。术中切除右上肺尖端结节1枚，大小约1.0 cm×1.2 cm×1.2 cm，未侵及脏层胸膜，未见肺门及纵隔淋巴结增大。术后石蜡病理提示，右上肺尖端结节原位腺癌(图 3)。患者病情好转出院。

**2:**
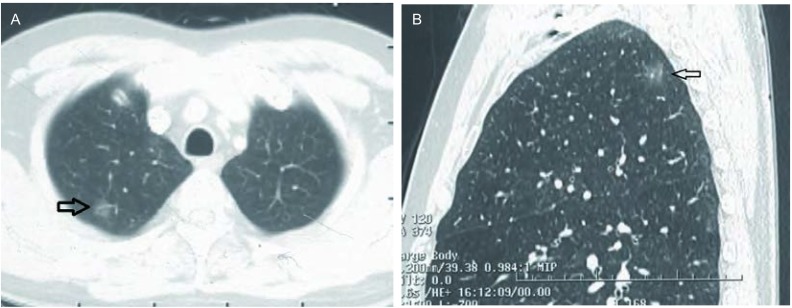
胸部CT检查。A：肺窗水平面显示右肺上叶尖端磨玻璃样结节（箭头所指）；B：矢状面显示右肺上叶尖端磨玻璃样结节（箭头所指）。 CT scan of chest. A: In lung window, horizontal, show the ground-glass nodule in apical segment of the superior lobe of the right lung (arrowhead); B: Sagittal, show the ground-glass nodule in apical segment of the superior lobe of the right lung (arrowhead).

**3:**
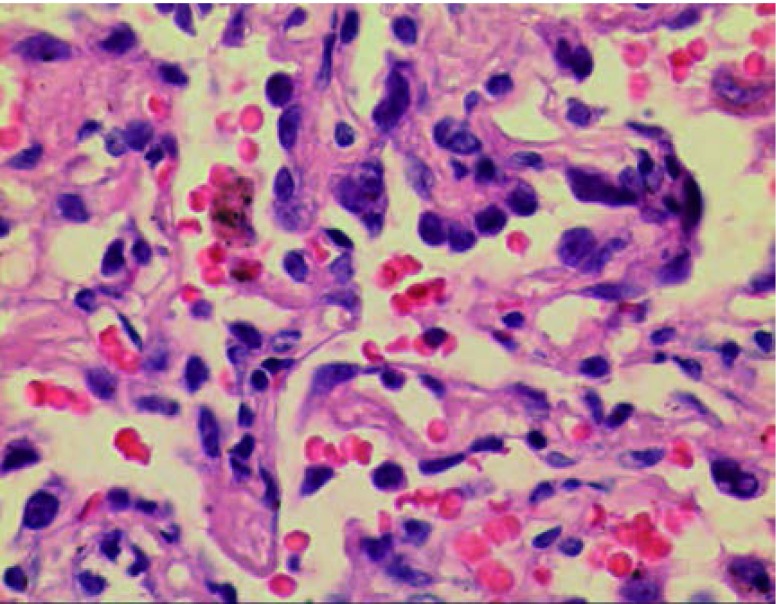
病理检查（×400）提示原位腺癌 Pathological image (×400): Adenocarcinoma *in situ*

## 2 讨论

随着CT设备分辨率的提高和普通人群体检意识的增强，越来越多的肺内非实性结节被发现，但其临床诊治流程仍有许多争议，一是认识不足，检查不到位，误诊漏诊较多；二是认识错误，造成过度检查、过度诊断及治疗。以肺部磨玻璃样影为CT表现的结节被称为磨玻璃样结节(ground-glass nodules, GGNs)可分为混合磨玻璃结节(mixed ground-glass nodule, mGGN)及纯磨玻璃结节(pure ground-glass nodule, pGGN)。多项研究^[3, 4]^证实非实性结节肺腺癌的CT表现和病理表现关系密切，例如，长期存在的，直径 < 5 mm的pGGN，其病理结果常表现为不典型腺瘤样增生^[5]^。同时，磨玻璃样结节中实性成分含量越多，预后越差^[6]^。在实际临床工作中，不同大小的pGGN，处理策略不同。有文献^[7]^指出，孤立的≤5 mm的pGGN，需采用连续薄层CT(1 mm)观察，排出将实行结节误诊为GGN的可能。此类GGN可能为偶发AHH，尽管AAH为癌前病变的一种，但AAH恶变时间仍未知，且孤立性AAH进展为浸润性腺癌的病例报道也很罕见。除此之外，在现有技术下精确测定≤5 mm病变的大小变化效果不理想，可重复性差^[8, 9]^。因此，为避免产生过多不确定的结论以及经济上的负担和过多的辐射伤害，目前，仍不建议常规CT随访这种病变^[10]^。孤立的> 5 mm的pGGN，发现后3个月进行CT复查以确定病变是否依然存在，而这期间无抗生素的使用指征^[11]^；如果病变仍然存在且无变化，则每年复查CT，至少随访3年。目前除手术切除尚无可靠的方法来明确病理诊断。因此，有研究^[12]^提出，对于直径> 8 mm的pGGN须行手术切除，而同时也有研究^[13]^表明20%的持续存在的pGGN为良性病变，因此对于pGGN的病理特点仍较大的争议。正因如此，密切观察此类GGN形态学的细微变化显得尤为重要，可以避免过度诊断及治疗^[2]^。然而，当此类pGGN病变大小超过10 mm，或患者具有肺癌病史，则被列为恶性肿瘤的高危因素，要予以重视。对于pGGN而言，PET/CT的诊断意义有限，无论性质如何，小的pGGN在PET上常不显示。而对此类病变行经皮细针穿刺活检结果准确度不高，容易导致误诊。Shimuzi等^[14]^在对 < 2 cm病变进行CT引导下经支气管肺穿刺活检的研究中发现GGN为主的病变诊断准确率只有51%；而对直径 < 1 cm的病变诊断准确率则更低只有35%。因此，对此类病变进行保守治疗时，经胸细针穿刺活检只适用于那些无法手术的病例。有研究报告^[12]^称，结合患者的年龄特征、病史、结节倍增时间等特点，对持续存在的直径>10 mm的pGGN须行手术切除，包括胸腔镜下外科楔形切除、肺段切除或亚段切除等。结合本例患者，右上肺叶尖段pGGN，直径> 10 mm，随诊1年余，结节持续存在，患者长期吸烟史，结合患者意愿，行胸腔镜下手术楔形切除病灶，术后病理为原位腺癌，最大程度保留患者肺功能，同时早期切除病灶，使患者受益。
